# Case Report: Pituitary metastasis as a presenting manifestation of silent gastric cardia adenocarcinoma

**DOI:** 10.3389/fonc.2022.1059361

**Published:** 2023-01-04

**Authors:** Andrea Ghezzi, Jessica Rossi, Francesco Cavallieri, Manuela Napoli, Rosario Pascarella, Romana Rizzi, Marco Russo, Gaetano Salomone, Antonio Romano, Corrado Iaccarino, Elisabetta Froio, Silvia Serra, Salvatore Cozzi, Lucia Giaccherini, Franco Valzania, Anna Pisanello

**Affiliations:** ^1^ Department of Biomedical, Metabolic, and Neural Sciences, University of Modena and Reggio Emilia, Modena, Italy; ^2^ Clinical and Experimental Medicine PhD Program, University of Modena and Reggio Emilia, Modena, Italy; ^3^ Neuromotor & Rehabilitation Department, Neurology Unit, Azienda USL-IRCCS of Reggio Emilia, Reggio Emilia, Italy; ^4^ Neuroradiology Service, Department of Diagnostic Imaging and Laboratory Medicine, Azienda USL-IRCCS of Reggio Emilia, Reggio Emilia, Italy; ^5^ Neurosurgery Unit, Neuromotor and Rehabilitation Department, Azienda USL-IRCCS of Reggio Emilia, Reggio Emilia, Italy; ^6^ Pathological Anatomy Service, Oncology Department and Advanced Technologies, Azienda USL-IRCCS of Reggio Emilia, Reggio Emilia, Italy; ^7^ Radiation Oncology Unit, Oncological Department and Advanced Technologies, Azienda USL-IRCCS of Reggio Emilia, Reggio Emilia, Italy

**Keywords:** pituitary metastasis, gastroesophageal junction adenocarcinoma, diabetes insipidus - neurogenic/central, hypopituitarism, visual disturbance

## Abstract

**Introduction:**

Pituitary metastases are very rare in cancer patients and often originate from lung or breast tumors. They usually occur in patients with known metastatic disease, but rarely may be the first presentation of the primary tumor.

**Methods:**

We present the case of a 58 years-old-man who reported a three-month history of polyuria-polydipsia syndrome, generalized asthenia, panhypopituitarism and bitemporal hemianopsia. Brain-MRI showed a voluminous pituitary mass causing posterior sellar enlargement and compression of the surrounding structures including pituitary stalk, optic chiasm, and optic nerves.

**Results:**

The patient underwent neurosurgical removal of the mass. Histological examination revealed a poorly differentiated adenocarcinoma of uncertain origin. A total body CT scan showed a mass in the left kidney that was subsequently removed. Histological features were consistent with a clear cell carcinoma. However, endoscopic examination of the digestive tract revealed an ulcerating and infiltrating adenocarcinoma of the gastric cardia. Total body PET/CT scan with 18F-FDG confirmed an isolated area of accumulation in the gastric cardia, with no hyperaccumulation at other sites.

**Conclusion:**

To the best of our knowledge, there are no reports of pituitary metastases from gastric cardia adenocarcinoma. Our patient presented with symptoms of sellar involvement and without evidence of other body metastases. Therefore, sudden onset of diabetes insipidus and visual deterioration should lead to the suspicion of a rapidly growing pituitary mass, which may be the presenting manifestation of a primary extracranial adenocarcinoma. Histological investigation of the pituitary mass can guide the diagnostic workup, which must however be complete.

## Introduction

Pituitary metastasis (PM) represents only the 0.4% of cerebral metastases and up to the 3.6% of the pituitary tumors. The most frequent primary localizations include breast, lung, kidney, and prostate tumors (1). To the best of our knowledge, there are no reports on pituitary metastases from a gastroesophageal junction (GEJ) adenocarcinoma. GEJ adenocarcinoma arises from the mucosa between 5 cm above and 5 cm below the proximal ends of the gastric folds and is classified by Siewert’s classification (2). Its incidence increased in the last decades, especially in western industrialized countries such as North America and Europe (especially in the Northern countries), whereas in Asia the incidence remains relatively low. This is probably due to lifestyle-related risk factors (3).

Here we describe the case of a patient in whom symptoms of pituitary metastasis represented the first manifestation of an occult GEJ adenocarcinoma.

## Case description

**Figure 1:**
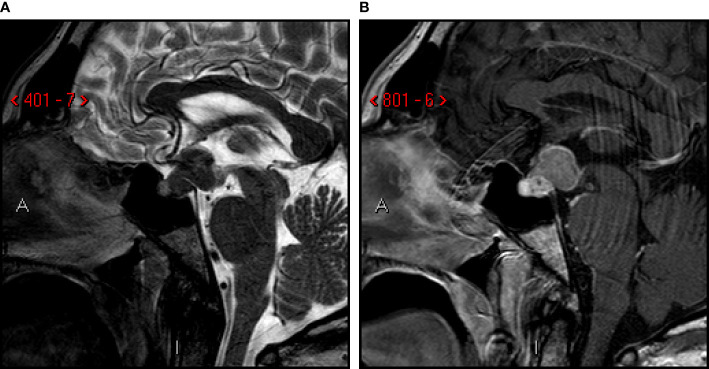
First MRI-scan. Sagittal T2-weighted **(A)** and post-contrast T1-weighted **(B)** images. Sellar and suprasellar mass involving posterior lobe and infundibulum of the pituitary gland, with low signal on T2-images. Compression of mammillary body, optic chiasm and tracts, third ventricle (infundibular recess).

A 59-year-old man came to our attention reporting a three-months history of progressive polyuria, polydipsia, fatigue, visual blurring, and dizziness. He was diagnosed with diabetes insipidus (DI) and panhypopituitarism and started replacement treatment with desmopressin, levothyroxine and cortisone acetate. His past medical history included the resection of multiple colic adenomas and a latent syphilis. He had no familiar history of cancer. He smoked cigarettes (47 pack/years) from the age of 12, and had an history of alcohol addiction, which he had quitted 15 years earlier. He underwent a brain MRI which showed a voluminous pituitary mass (18.5x19.5x15.5 mm diameter; **Figure 1**) involving the posterior lobe and the pituitary stalk with suprasellar invasion, causing posterior sellar enlargement, and compression of the surrounding structures including the optic chiasm and the optic tracts, with loss of neurohypophysis signal. These findings were consistent in first hypothesis with a primary pituitary tumor or craniopharyngioma.

An endoscopic endonasal multidisciplinary debulking surgery was performed by a combined approach with an otolaryngologist and a neurosurgeon. The procedure was performed under general anesthesia and preoperative antibiotics administration (cefazolin). Intraoperative magnetic neuronavigation with S7 StealthStation neuronavigation AxiEMTM ENT system (Medtronic, Inc.) is routinely used during this procedure. The patient’s head is placed in a horseshoe headrest, slightly elevated, in extension, and slightly turned to the right (towards the operator). Following Povidon-Iodine disinfection, nasal infiltration with local anesthetic and adrenaline solution, with a rigid 0° endoscope (Karl Storz GmbH, Tuttlingen, Germany) a right middle turbinectomy is performed. A pedicled septal mucosa flap was prepared for final closure, and a large anterior sphenoidotomy and posterior septotomy was performed. After drilling planum sphenoidale, the optic carotid junction was exposed on both sides by high-speed drilling of the medial carotid optic processes.

Thus, through a “T shape” incision of the planum and sellar dura, the arachnoid was opened to gain access to the suprasellar lesion with a reddish gray fleshy appearance without any cleavage plane with the optic chiasm and the pituitary stalk. Several lesional samples were carried out suggesting the intraoperative diagnosis of probable metastatic malignant lesion. Therefore, a limited decompressive surgery of the optical pathways using an ultrasonic aspirator (CUSA) was decided as the main surgical strategy. Our closure technique is provided by a free flap of autologous abdominal fascia inlay and overlay of the previously prepared flap of the pedunculated septal mucosa.

**Figure 2:**
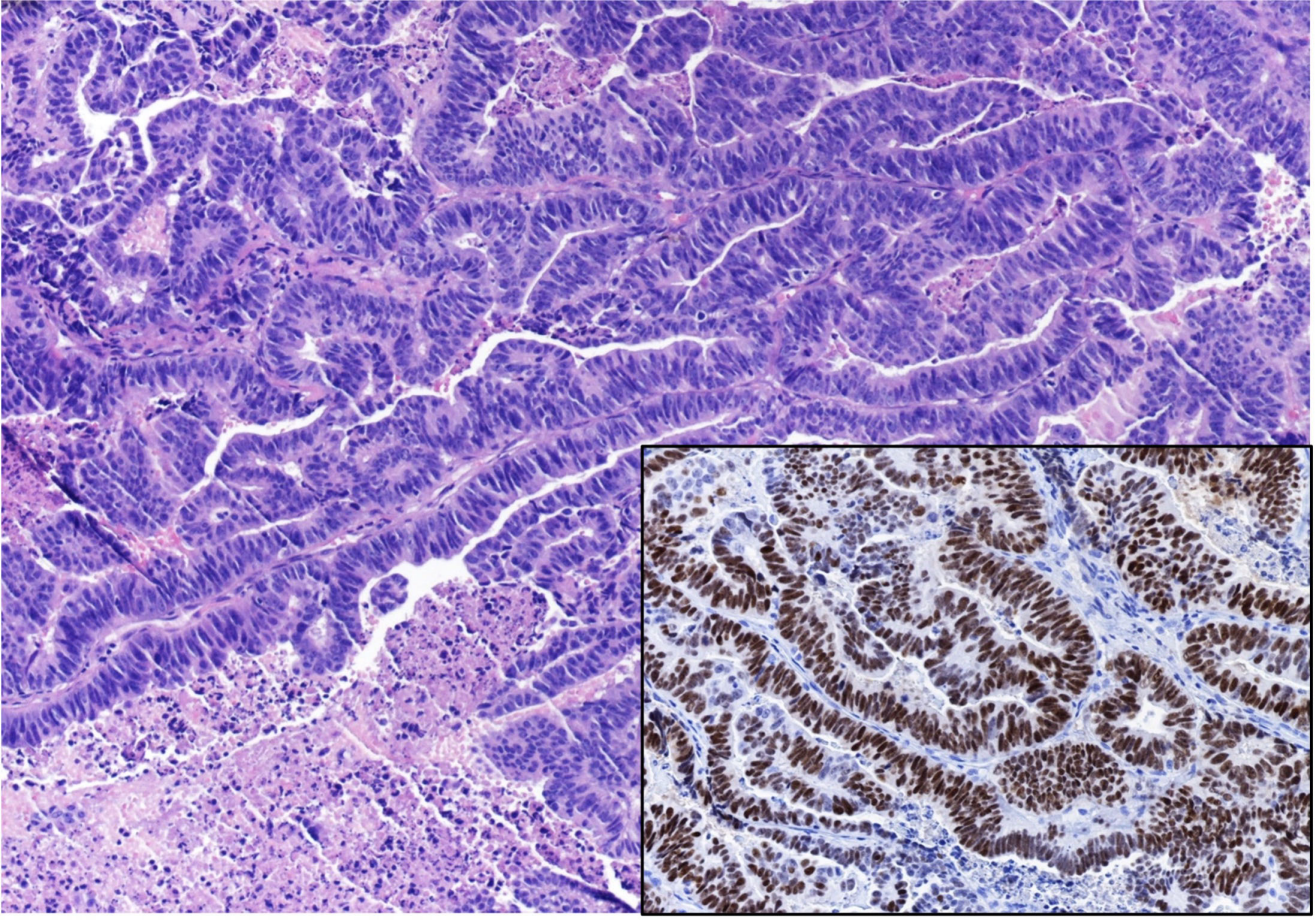
Histopathological examination of the sellar mass, consistent with a pituitary localization of an Adenocarcinoma, showing a pseudopapillary and glandular architectural pattern with necrosis and brisk mitotic activity (EE 20x). Insert: Immunostaining for CDX2 (20x).

Subsequent histologic examination showed a poorly differentiated HER-2-positive adenocarcinoma of uncertain origin (**Figure 2**). Neoplastic cells showed marked atypia, areas of necrosis and high mitotic index. At the immunohistochemical staining, the neoplasm was positive for CDX2, keratin 7, keratin 20, keratin 19, and negative for PAX8, p63, TTF1 and GATA 3.

A total-body CT scan showed a mass in the left kidney that was subsequently removed. Histological features were consistent with a clear cell carcinoma. However, endoscopic examination of the digestive tract revealed an ulcerating and infiltrating adenocarcinoma of the gastric cardia. Total body PET/CT scan with 18F-FDG confirmed an isolated area of accumulation in the gastric cardia, with no hyperaccumulation at other sites. The patient was discharged with an indication for oncology care. At the time of discharge, neurologic examination showed bitemporal hemianopsia and hyposmia, and Karnofsky Performance Status (KPS) score was 80.

**Figure 3:**
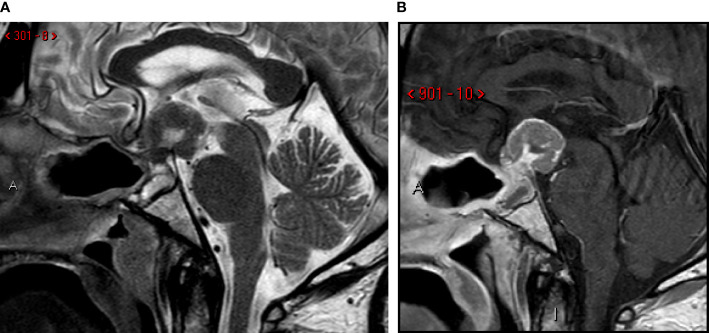
One-month follow-up MRI scan. Sagittal T2-weighted **(A)** and post-contrast T1-weighted **(B)** images. Post-surgical sellar modification with increase of the residual tumor in suprasellar spaces, third ventricle invasion and mammillary body compression and infiltration.

**Figure 4:**
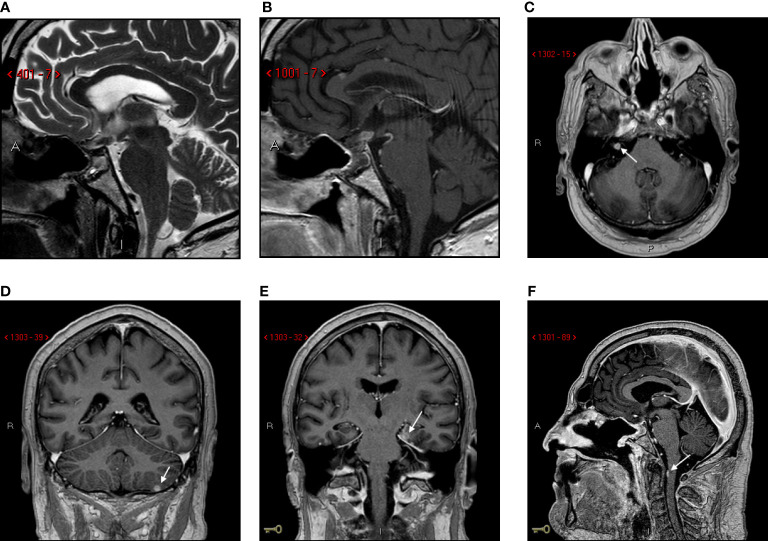
Follow-up MRI scan at 139 days. Sagittal T2-Weighted **(A)** and post-contrast T1-weighted **(B)** images. Post-surgical sellar modification with small residual tumor in suprasellar/infundibular region. Axial post-contrast T1-weighted images **(C-F)**: multifocal leptomeningeal dissemination (arrows).

One month later, the patient was readmitted because of visual symptoms worsening. A new brain MRI showed an extension of the post-surgical residual tumor, with compression of optic chiasma, hypothalamus, mammillary bodies, and third ventricle (**Figure 3**). Therefore, 36 days after surgery, adjuvant radiation therapy was started (single cycle of 5 sessions, 6 Gy for every session, total dose 30 Gy) on the post-surgical residual, followed by six cycles of chemotherapy with cisplatin (115 mg, started 9 days after the end of radiation therapy and converted to carboplatin 400 mg for the last three cycles after the nephrectomy), 5-fluorouracyle (4000 mg/m2) and trastuzumab (6 mg/m2). A follow-up MRI study was performed after 139 days that showed multifocal leptomeningeal dissemination in the posterior cranial fossa (**Figure 4**).

The patient died 11 months after the onset of the symptoms because of the cranial dissemination of the tumor.

## Discussion

Pituitary metastasis is a rare but increasing condition, as survival in patients with cancer has been improving in recent years. Common reported sites of primary tumors are breast (37.2%), lung (24.2%), renal cell (5%) and prostate (5%) (4).

The most common sites of GEJ tumor metastasis are lung and liver (5). To the best of our knowledge, this is the first case of pituitary metastasis from gastroesophageal junction (GEJ) adenocarcinoma.

Moreover, whereas the main symptoms in patients with GEJ tumors are dysphagia and weight loss (6), our patient manifested signs of a rapid enlarging sellar mass, without any symptom of gastric or systemic involvement. Diabetes insipidus is one of the most common symptoms in PM (27.4%), together with visual disturbance (generally bitemporal hemianopsia), fatigue, headache, cranial nerve palsies and anterior pituitary deficiencies such as hypothyroidism and hypocortisolism (7, 8). The frequency of diabetes insipidus in pituitary metastases reflects their predominance in the neurohypophysis, which is due to the lack of direct arterial blood supply to the adenohypophysis, that is nourished through the hypophyseal portal system (8, 9). Conversely, diabetes insipidus is found in only 1% of pituitary adenomas (9) making it a good sign to differentiate between PM and adenomas (9).

Radiologic findings of PM are mostly unspecific. The brain MRI usually shows an isointense or hypointense mass on T1w images and an hyperintense signal in T2w images, with a homogeneous contrast enhancement (9). A mass effect on the third ventricle might cause an hyperintense T2-weighted signal on adjacent brain parenchyma because of the vasogenic edema, as it was the case in our patient. Radiological differential diagnosis of intra- and suprasellar lesions includes: pituitary adenoma, lymphocytic hypophysitis, craniopharyngioma and pituicytoma (10). Lymphocytic hypophysitis in an idiopathic inflammation usually involving the anterior portion of pituitary gland or stalk and is more frequently observed in pregnant or postpartum females (10). Pituicytoma is a rare tumor arising from pituicyte, a specialized glial cell in neurohypophisis and infundibulum, and is difficult to be distinguished from a metastasis if primary tumor is unkown (10). In contrast to primitive pituitary tumors, in particular adenoma and craniopharyngioma, metastases can be distinguished through some characteristic features such a dumbbell shape, an indentation of the diaphragma sellae, sellar erosion without enlargement (due to the rapid growth of metastases versus the remodeling of slow-growing pituitary adenomas) and loss of the posterior bright spot (1, 9). However, radiological evaluation is generally not sufficient to distinguish PM from other lesions. Therefore, histologic examination is crucial, especially in doubtful cases, as in our patient, in whom instrumental investigations did not document other systemic metastases but confirmed the presence of a double pathology (a clear cell renal carcinoma and a gastric cardia adenocarcinoma). Prognosis of patients with PM is unfavorable and depend mostly on aggressiveness of the primary neoplasia (9). The mean overall survival is about 6–7 months (9), as in our case, although most patients die a few months after diagnosis.

We performed research in PubMed, looking for other cases of pituitary metastases and we found 107 cases between case series and single case reports (11–30-33). We found only 7 cases of pituitary metastasis originating from gastroenteric tract (12, 25, 29, 31, 32) and no cases of pituitary metastasis from gastro-esophageal junction adenocarcinoma (**Supplementary Table 1**).

## Conclusions

The pituitary gland can be a site for metastases in patients with gastric cardia adenocarcinoma. Sudden onset of diabetes insipidus in a patient over 50 years of age should always raise the suspicion for PM, regardless of a malignancy history (9). In patients with limited metastatic disease, as well as double pathology, surgery and histological examination are essential to identify the primary tumor and better guide the definition of the prognosis and the most appropriate therapy.

## Data availability statement

The original contributions presented in the study are included in the article/**Supplementary Material**. Further inquiries can be directed to the corresponding author.

## Ethics statement

The studies involving human participants were reviewed and approved by the Ethics Committee of Area Vasta Emilia Nord. The patients/participants provided their written informed consent to participate in this study. Written informed consent was obtained from the individual(s) for the publication of any potentially identifiable images or data included in this article.

## Author contributions

AG, JR and FC contributed to writing this article and to the literature review. MN and RP provided the neuroradiological images and descriptions. EF and SS provided the pathological images, descriptions and information regarding histological examination. RR, MR, GS, FV and AP contributed with information about the patient’s clinical course. AR and CI furnished data regarding surgical procedures. SC and LG contributed with details on radiation therapy. All authors contributed to the article and approved the submitted version.
