# Supplementary material for: Case Report: Pituitary metastasis as a presenting manifestation of silent gastric cardia adenocarcinoma
Source: Front Oncol. 2023 Jan 4;12:1059361. doi: 10.3389/fonc.2022.1059361 (PMC9846627; doi:10.3389/fonc.2022.1059361)
Supplement: Supplementary file 1 [file Table_1.docx]

| Author, year of publication | N° of patients | Primary tumor | Clinical presentation | Presence of DI | Hypopituitarism | First MRI findings |
| --- | --- | --- | --- | --- | --- | --- |
| Lithgow et al, 2020^12^ | 18 | Lung (7, 39%)  Breast (6, 33%)  Prostate (2, 11%)  Melanoma (2, 11%)  Renal (1, 6%) | Visual field defects (7)  Cranial nerve III and/or IV palsy (4)  Symptoms/signs of cortisol deficiency (2)  Loss of VA (2)  Symptoms unrelated to pituitary metastases (abnormal sensation in right side of the face, change in left hand function; ataxia and vomiting; unsteady gait; facial palsy; (4)  Incidental finding (2) | 3/18 | N/A | N/A |
| Schill F et al, 2019^13^ | 38 | Breast: 17 (45%) Lung: 8 (21%) Prostate: 4 (11%) Esophagus: 1 (3%) Endometrium: 1 (3%) Renal: 1 (3%) Submandibular gland: 1 (3%) CNS lymphoma: 1 (3%) AML: 1 (3%) Unknown: 3 (3%) | Headache (20)  Visual impairment (16)  Nausea/vomiting (11)  Diplopia (10)  Weight loss (5)  Vertigo (5) | 10/38 | N/A | Suprasellar extension (74%, in 64% of which it was the only direction of growth) with 3 different patterns:   1. extension along the pituitary stalk with a dumbbell shape due to constriction by the sellar diaphragm; 2. asymmetrical, lobular growth along the pituitary stalk, the upper surface of the diaphragma sellae, and the dura mater lining the tuberculum sellae; 3. characteristics indistinguishable from a typical benign adenoma expanding the diaphragm rather than being constricted by it.   Parasellar and/ or infrasellar growth (36%).  Bone resorption without remodeling (29%).  Skeletal erosions (40%), observed in 50% of PM from lung cancers and 24% of PM from breast cancers. |
| Gandhi et al, 2020^14^ | 1 | Renal cell carcinoma | Diplopia, headache | No |  | Heterogeneous sellar mass invading the right cavernous sinus with a leftward deviation of the infundibulum. Small amount suprasellar extension with mild mass effect on the optic chiasm |
| [Parthasarathy](https://pubmed.ncbi.nlm.nih.gov/?term=Parthasarathy%20S%5BAuthor%5D) S, 2022^15^ | 1 | Breast | Confusion secondary to hyponatriemia | Yes | Yes | Well-circumscribed, heterogeneously enhancing sellar mass with suprasellar extension, involvement of the infundibulum and hypothalamus, and mass effect on the optic chiasm. |
| Bailey D et al, 2021^16^ | 1 | Urothelial carcinoma | Altered mental status, poor oral intake, and falls for one week. | Yes | Yes | Lesion involving the sella turcica with heterogeneous signal intensity and enhancement. |
| Oueslati I et al, 2020^17^ | 1 | Breast | Weight loss, nausea and vomiting, headache, blurred vision, and decreased visual acuity. | No | Yes | Large heterogeneous mass involving the pituitary anterior lobe, isointense on both T1- and T2-weighted images. Intensive homogeneous enhancement after gadolinium. Extension into the optic chiasm, left cavernous sinus, and sphenoid sinus. Normal posterior lobe bright spot. |
| Chun-Yang L et al, 2021^18^ | 1 | Lung adenocarcinoma | Bilateral blurred vision, dizziness, polyuria, nocturia, severe fatigue, somnolence, decreased libido, intermittent nausea and vomiting. | Yes | Yes | A mass protruding from the sella turcica and sphenoid sinus. Iso- and spot-like high and low intensity signals on T1-weighted images and high, slightly high, and low intensity signals on T2-weighted images. Heterogenous enhancement. Optic nerve and papillary body surrounded by the mass. Mass protrusion into the third ventricle and invasion of the bilateral cavernous sinus and internal carotid artery. |
| Watanabe N et al, 2020^19^ | 1 | Lung adenocarcinoma | Anorexia, hyponatriemia | Yes | Yes | Dumbbell-type pituitary tumor with suprasellar extension. Contrast-enhancement after gadolinium injection. |
| El Habnouny J et al, 2020^20^ | 1 | Breast | Headache, palpebral ptosis due to third cranial nerve palsy | No | Yes | Intra- and suprasellar lesion lateralized to the right, initially suggesting a macroadenoma, with extensive infiltration of the right cavernous sinus up to the hard external dura mother, erosion of the antero-inferior sellar floor, and massive infiltration of the right cavernous sinus. |
| Moon RDC et al, 2021^21^ | 1 | Renal cell carcinoma | Headache, ataxia | No | Yes | Large sellar mass with contrast enhancement. Suprasellar tumor extension with displacement of the optic chiasm and parasellar extension to the anterior cerebral and anterior communicating arteries. Clival invasion, prominent vascular flow voids on T2-weighted images. |
| Ng S et al, 2020^22^ | 1 | Melanoma | Visual field defect | No | Yes | Sellar lesion with suprasellar expansion leading to a compression of the optic chiasm. Heterogeneous high signal intensity on T1-weighted images, mild homogeneous enhancement on T1-weighted images with gadolinium infusion, and low signal intensity on T2-weighted images. |
| Ahmad S et al, 2020^23^ | 1 | Prostate | Headache, cranial nerve III palsy | No | No | Sellar mass invading the right cavernous sinus, displacing the pituitary stalk to the left. |
| Du H et al, 2020^24^ | 1 | Endometrial adenocarcinoma | Polyuria, polydipsia, weight loss, fatigue | Yes | No | Thickening of the pituitary stalk, involvement of the superior border of the pituitary gland, and disappearance of the hyperintensity in the posterior lobe in T1-weighted images. Significant and continuous contrast enhancement of the pituitary stalk, and heterogeneous enhancement of the pituitary gland. |
| Sheahan et al, 2020^25^ | 1 | Lung adenocarcinoma | Right eye blindness, headache, dizziness, nausea, amenorrhea | No | Yes | Sellar and suprasellar mass with right cavernous sinus invasion. |
| Estrada A et al, 2019^26^ | 2 | Papillary thyroid cancer | Progressive weakness, fatigue, decreased appetite | No | Yes | Infiltrative process enlarging the pituitary gland, extending through the stalk and into the hypothalamus. Other cerebral metastatic lesions. |
|  |  | Colon adenocarcinoma | Excessive thirst, increased urinary frequency, nocturia | Yes | No | Infiltrative process within the pituitary gland, stalk, and hypothalamus. |
| Gao H et al, 2019^27^ | 1 | Follicular thyroid carcinoma | Headache, pain to the right eye | No | No | Pituitary lesion, isointense on T1- and T2-weighted images, contrast enhanced, compressing the optic chiasm and invading the cavernous sinus. |
| Nose K et al, 2019^28^ | 1 | Breast cancer | Headache, cranial nerve III palsy, loss of visual acuity, anorexia, general malaise | No | Yes | Heterogeneously enhanced lesion of the pituitary gland with thickened pituitary stalk. Dura mater thickening around the anterior processus clinoideus (dural tail). |
| Shen Z et al, 2018^29^ | 1 | Renal cell carcinoma | Headache, nasal congestion, nausea, vomiting, right eye visual acuity decline | No | N/A | Invasive sellar mass with cavernous sinus and nasal cavity extension. |
| Javanbakht A et ak, 2018^30^ | 11 | Breast (2)  Testicular germ cell tumor (1)  Lung carcinoid (1)  Colon adenocarcinoma (1)  Yolk sac tumor (1)  Papillary thyroid cancer (1)  Hodgkin lymphoma (1)  Diffue large B cell lymphoma (1)  Eosinophilic granuloma (1)  Multiple myeloma (1) | DI (3)  Panhypopituitarism (3)  Visual involvement (2) | 3/11 | N/A | N/A |
| Souza Mota J et al, 2018^31^ | 1 | Follicular thyroid carcinoma | Headache, retroorbital pain, left visual acuity worsening, and ptosis | No | Yes | Enlarged suprasellar formation with an intense contrast enhancement, compromising the left cavernous sinus. |
| Castle‑Kirszbaum M et al, 2018^32^ | 12 | Breast (4, 33%)  Lung (4, 33%, of which 3 NSCLC and 1 SCLC)  Plasmocytoma (1, 8%)  Melanoma (1, 8%)  Oesophageal carcinoma (1, 8%)  Colorectal carcinoma (1, 8%) | Symptoms of hypopituitarism (10, 83%)  Symptoms of DI (9, 75%)  Headache (8/12, 67%)  Visual field defect (8/12, 67%)  Cranial nerve III palsy (3, 25%) | 9/12 | 10/12 | Contrast-enhancing sellar lesion with suprasellar extension (83%), infundibulum extension (25%), compression/displacement of the optic chiasma (42%), sellar floor erosion (33%). |
| Zhao Y et al, 2018^33^ | 10 | SCLC (2, 20%)  Renal cell carcinoma (2, 20%)  Large cell lung carcinoma (1, 10%)  Pulmonary adenocarcinoma (1, 10%)  Medullary thyroid carcinoma (1, 10%)  Rectal cancer (1, 10%)  Small intestinal leiomyosarcoma (1, 10%)  Stomach cancer (1, 10%) | Visual impairment (5, 50%)  Cranial nerve III palsy (4, 40%)  Gait disturbances (2, 20%)  Symptoms of DI (2, 20%)  Central obesity (1, 10%) | 2/10 | 8/10 | Sellar-occupying lesions with homogeneous (22%) or heterogeneous (78%) enhancement* |

Table 1. Summary of case reports and case series about pituitary metastasis. Abbreviations: AML= acute myeloid leukemia; CNS= central nervous system; DI= Diabetes Insipidus; N/A= Not Applicable; PM= pituitary metastasis; SCLC: Small Cell Lung Carcinoma; VA: Visual Acuity.

*extra-sellar involvement not specified
